# Photobiomodulation therapy for the management of recurrent aphthous stomatitis in children: clinical effectiveness and parental satisfaction

**DOI:** 10.4317/medoral.23573

**Published:** 2020-05-10

**Authors:** Elena Bardellini, Federica Veneri, Francesca Amadori, Giulio Conti, Alessandra Majorana

**Affiliations:** 1 University of Brescia, Department of Oral Medicine, Dental Clinic, P.le Spedali Civili di Brescia, Italy; 2 Department of Oral Surgery, University Vita-Salute San Raffaele, Milan, Italy

## Abstract

**Background:**

This study aims to evaluate the effectiveness of the photobiomodulation therapy (PBMT) in the treatment of minor recurrent aphthous stomatitis (MiRAS) in children, in terms of pain relief, lesion size reduction and the parental satisfaction of the therapy.

**Material and Methods:**

This randomized controlled study was carried out on 60 children with clinical diagnosis of MiRAS. Patients were randomized into two groups: group A receiving laser therapy and group B receiving sham therapy (placebo). Laser therapy (diode laser, λ: 645 nm) was administered on day 1 (T0) for three consecutive days. Patients were evaluated also on day 4 (T1), on day 7 (T2) and on day 10 (T3). Oral aphthous lesions size was assessed through a periodontal probe to measure the diameter length (mm); pain was evaluated through the Visual Analogue Scale (VAS); parental satisfaction was assessed through a questionnaire.

**Results:**

The difference in the reduction of ulcers diameters between the two groups resulted statistically significant at T1 and at T2 (*p*<0.05). A statistically significant difference in pain reduction between two groups was found at T1 (*p*<0.05). No statistically significant difference between the two groups of parents was found as concerns the parental acceptance of the procedure and the discomfort for the need of multiple appointments.

**Conclusions:**

PBMT is to be considered effective in the treatment of MiRAS in children and well- accepted by the parents of the children themselves.

** Key words:**Ulcers, children, lasers

## Introduction

Recurrent aphthous stomatitis (RAS), with its frequency of 5-25%, is one of the most common oral mucosal disorders characterized by painful, recurrent single or multiple small oval or round ulcerations with well-defined edges, having yellow or grey background with surrounding erythematous haloes (1). It can affect both adults and children (2).

Based on size, number and duration of the lesions, it can be classified into three types: minor (MiRAS), major (MjRAS), and herpetiformis ulcers (HUs) (3-4).

MiRAS, accounting for more than 80%–90% of RAS cases, typically shows lesions of less than 1 cm in diameter and heals within 7–14 days without scar formation. MjRAS lesions exceed 1 cm in diameter and heal within 20–30 days with scarring. HUs are characterized by 1–3 mm, multiple and clustered lesions, which may coalesce into larger ulcers and take up to 15 days to heal (3-4).

RAS etiology is multifactorial, including immune system dysfunction, genetic factors, food allergens, nutrition deficiency, hormonal changes, local trauma, endocrine alterations, stress, chemical products and microbial agents (3-8).

A variety of treatments have been proposed over the last years, including topical anesthetics, analgesics, antiseptics, topical steroids, immunomodulating drugs, mucosal barrier gel, sucralfate and herbal remedies (7-13).

Although the ulcers are usually self-limiting, these lesions are particularly challenging in children as their debilitating nature may interfere with daily activities such as eating, swallowing and speaking (14). Children are also more prone to super-infections and hyper-salivation and they may also suffer from lack of appetite, fatigue, difficulties in concentration and nervousness. In pediatric age it is therefore important to reduce symptoms and to accelerate the healing process.

The use of biostimulating lasers has recently proven useful in several medical fields for treating chronic and acute pain conditions thanks to its anti-inflammatory and analgesic effects. In fact, it promotes re-epithelialization, fibroblasts proliferation, collagen synthesis, it increases vascularity and decreases the alterations in nerve impulse conduction (14).

In a recent study, Rocca *et al*. (15) analysed the effect of the laser treatment of aphthous lesions in adult patients with four devices available on the market, two with wavelength in the infra-red region (2940 nm 808 nm) and two with a wavelength in the visible region (450 nm and 645 nm). Diode lasers 808 nm and 450 nm showed similar results with an improvement starting already after the application and gradually improving until 7 days after treatment without any statistically significant difference between them. Diode 645 nm was the device gaining the earliest effect reducing the pain already during the treatment and maintaining it at low level immediately after the laser application.

The aim of this study is to evaluate the efficacy of PBMT with a 645 nm diode laser in the treatment MiRAS in pediatric patients in terms of pain relief, lesions diameters reduction and parental satisfaction of the therapy.

Materials and Methods

- Sample Selection

This randomized controlled study was carried out on a group of consecutive children (aged 5 to 12 years old) with clinical diagnosis of MiRAS ulcers referred to the Pediatric Dentistry Department of the Dental Clinic of Brescia (Italy). Enrollment criteria were clinical diagnosis of MiRAS according to the Stanley classification (ulcers less than 10 mm in size, sited on nonkeratinized mucosal surfaces, recurring at intervals of 1-4 months, healing in 7-10 days) (16). Exclusion criteria were chronic diseases (e.g. coeliac disease, IgA deficit, diabetes, etc.) and drug intake (e.g. antibiotics, antifungals, corticosteroids, hormone therapies, etc.).

Patients were randomized into two groups by a computer code: group A which included patients receiving laser therapy and group B receiving sham therapy (placebo), i.e. the device was switched on but the hand piece did not work. Randomization was performed using an automatically generated list in a 1:1 block size for two patients. Patients included in the study were randomly assigned to one of the 2 groups. Operators who performed the treatment were not blinded to the allocation group. The blindness of enrolled subjects was guaranteed by the sham treatment, which was indistinguishable from PBMT. Outcome evaluators were also blinded to the study group.

Dentist, patients and caregivers wore appropriate protective eyewear, following international safety procedures. The patients and their caregivers were advised not to take any topical or systemic medications or products from day 0 to day 7. Laser therapy was administered on day 1 (T0) for three consecutive days. Pain and diameters of the lesions were assessed on day 1 (T0), on day 4 (T1), on day 7 (T2) and on day 10 (T3).

- Laser equipment

The laser device used for this trial was a diode laser (RAFFAELLO 980 BIO - Dental Medical Technologies – DMT S.r.l.). PBMT was administered by a trained dentist and irradiated in the sites of oral aphthous lesions with 645 nm wavelength, power 100 mW, spot size 1 cm2, 30 s per cm2, energy density 10 J/cm2, continuous mode.

- Sham treatment 

Patients received the exact repetition of the treatment modality but without any laser emission: although switched off, the laser devices emitted the same sound and showed the same screen parameters when working in the effective PBMT modality.

- Aphthous ulcerations diameters

Oral Aphthous lesions were measured through a periodontal probe to score the diameter length (mm). The scoring was performed on day 1 (immediately before the beginning of treatment) (T0), on day 4 (T1), on day 7 (T2) and on day 10 (T3) as follow-up by three calibrated clinicians. The clinicians who prescribed the therapy were not involved in lesion measurement.

- Pain Scoring

Pain was evaluated through the Visual Analogue Scale (VAS) at the same timing of lesion measurement. According to this system, 1 indicates no pain and 10 indicates severe pain; patients were asked to select a number from 1 to 10 on a ruler with icons faces to express the intensity of their pain.

- Parental acceptance of the therapy

A questionnaire was handed out at T2 to evaluate the parental acceptance of the PBMT. The questionnaire used to examine the parental acceptance of PBMT was written in Italian language and consisted of questions divided into 2 parts. The first part of the survey included demographic information of the parents while in the second part of the survey the parents were asked to rate their acceptance of the laser therapy and the discomfort for the need of multiple appointments. The rating was determined by a five-point scale ranging from 1 (unsatisfied) to 5 (very satisfied). The parents answered the survey in the waiting room and they were allowed sufficient time to answer the questionnaire alone without any explanation of the examiner.

- Statistical Analysis

Statistical analysis was descriptive, including mean, standard deviation and percentiles for variables such as sex, age and localization. To compare the two groups, we analysed the data on gender, disease and parents acceptance as frequencies and percentages. Concordance or differences in the frequency distribution between the two groups were tested using the Exact Fisher’s test. Student t test was used to compare VAS and size between groups. A level of significance of 5 % was used and data were analysed using Stata® software for Mac.

Estimating that there is a success percentage of 90 % on day 3 for the group treated through PBMT by laser and of 50 % for the control group, the minimum number of patients for the study, assuming alpha 0.05 and beta 0.20 (study power = 80 %), was calculated to be 40 (at least 20 per group).

## Results

A total of 60 children (37 females and 23 males) with MiRAS were included in the study according to the enrollment criteria. Size of the lesions ranged from 5 mm to 8 mm. The involved oral sites were: upper and lower lip, buccal mucosa and soft palate.

Demographic and clinical features are summarized in Table 1. The number of lesions and the lesion diameters before therapy were similar for the two treatment groups (*p*>0.05).

- Lesions diameters

At T0, the mean of lesion diameters was 6.82±1.52 mm for group A and 6.34±1.77 mm for group B. On the fourth day (T1), the mean decreased to 3.82±2.02mm for group A and 4.79± 1.60 mm for group B; after seven days (T2), the mean resulted 2.40±0.5 mm in group A and 3.20±0.5mm in group B. Both groups showed a progressive reduction in ulcer extension, with a complete healing on day 10. The difference in the reduction of ulcer diameters between the two groups resulted statistically significant at T1 and at T2 (Table 2).

- Pain evaluation

The medians of VAS at the three intervals and *p-value*s are displayed in Table 2. A statistically significant difference in pain reduction between two groups resulted only at T1 (*p*<0.05). No pain was reported after 10 days from the beginning of the treatment.

- Parental acceptance of the procedure

No statistically significant difference between the two groups of parents was found concerning parental acceptance of the procedure and the discomfort for the need of multiple appointments (Table 3).

**Table 1:**
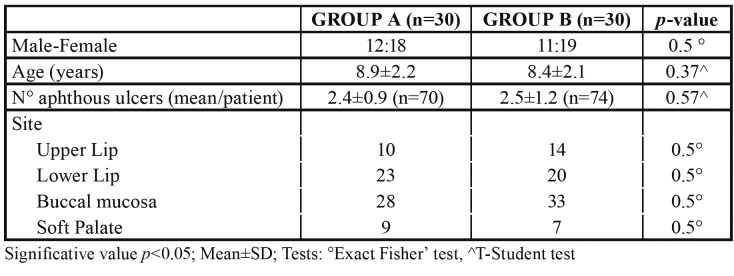
Demographic characteristics of the children.

**Table 2:**
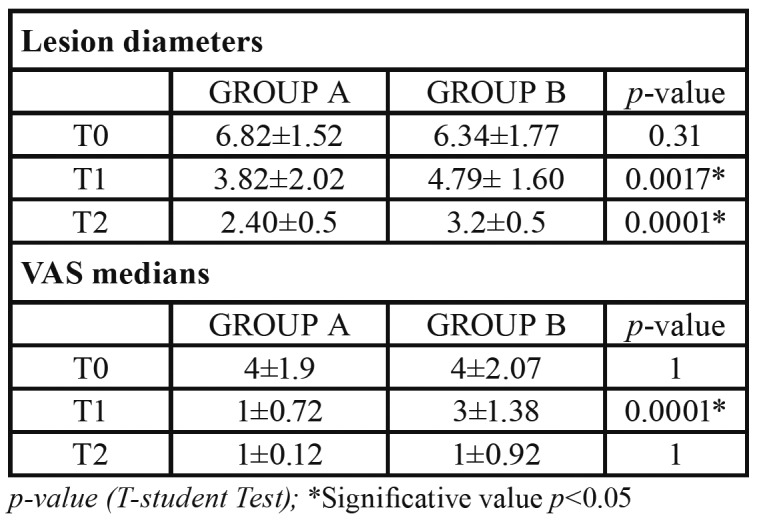
Lesion diameters and VAS medians at T0, T1, T2.

**Table 3:**
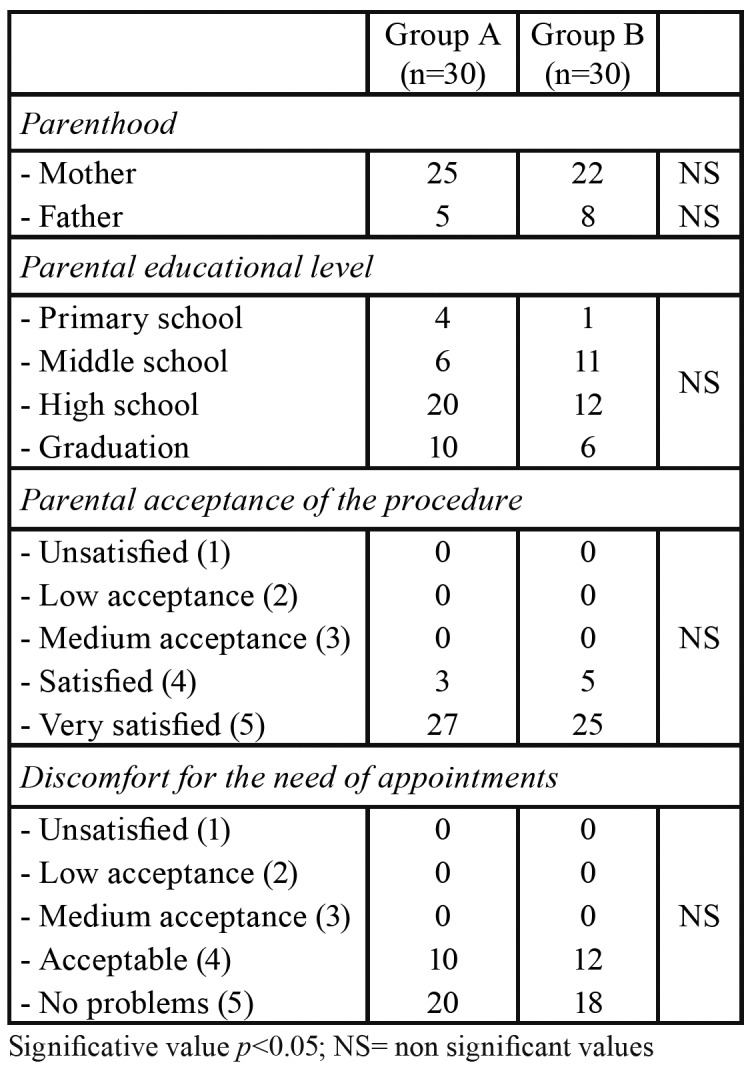
Demographic and educational characteristics of the parents, levels of acceptance of the procedure and discomfort for the needs of multiple appointments.

## Discussion

Minor RAS is a common painful inflammatory condition characterized by recurrent ulcers with surrounding inflammatory halos; the underlying etiology remains unclear and a standardized treatment is still not well defined (1). According to a recent review (17), the Authors suggest identifying and managing the main predisposing factors (such as vitamin deficiencies, coeliac disease, anemia) by a detailed clinical history and laboratory test. Topical medications appear to be the first choice of treatment for MiiRAS. Such preparations do have limitations with respect to drug delivery, subsequent compliance and retention on the oral mucosa (18).

In the present study, the efficacy of the use of photobiomodulation therapy (PBMT) in the treatment of MiRAS pediatric patients was evaluated, both in terms of pain relief and lesions size reduction. From our results, the use of a diode laser with 645 nm wavelengths allowed a statistically significant reduction of the ulcers diameters at T1 and at T2. This finding is in agreement with a recent randomized clinical trial in adults that reported a statistically significant reduction in lesion sizes at each follow-up time, especially on day 3 (19).

According to our results, PBMT determines a significant pain reduction after three days of therapy (T1). This is in agreement with Aggarwal *et al*. (19), who reported a statistically significant reduction in pain by using a diode laser (810nm) when compared to the sham therapy group. Lalabonova and Daskalov (20) also reported a statistically significant reduction in erythema dynamics and epithelisation time in the laser therapy group.

Even if the exact mechanisms through which the laser induces pain relief are still not clear, it has been demonstrated that laser light has three main effects: analgesia, anti-inflammation and promotion of wound healing. During its interactions with biological tissues, laser energy is converted into energy useful to cells; it induces an increase in ATP mitochondrial production, serotonin and endorphins release (21,22). Moreover, local blood circulation, cellular proliferation and protein synthesis are increased. It is known that anti-inflammation and analgesia are connected to both an increase of peripheral endogenous opioids and a decrease of pro-inflammatory cytokines and free oxygen radicals (21,22).

The parents of both patients in laser therapy as well as those in sham therapy well accepted the laser procedure and did not complain of any problems regarding the time spent for multiple appointments. This is probably due to the fact that the parents of children with canker sores consider these debilitating lesions deserving of any attempt to alleviate symptoms.

The encouraging results of our investigation support the choice of PBMT for treating MiRAS in children. Previous studies in adults have been conducted, investigating the influence of varying laser device parameters such as power, wavelengths and number of sessions, but in general PBMT resulted in a significant improvement of the disease. More RCTs studies are needed to define specific device parameters and protocols to be applied in the everyday clinical management of MiRAS in paediatric patients.
